# Supplementary material for: Activity of glucose-6-phosphate dehydrogenease and its correlation with inflammatory factors in diabetic retinopathy
Source: PLoS One. 2024 Dec 2;19(12):e0312452. doi: 10.1371/journal.pone.0312452 (PMC11611225; doi:10.1371/journal.pone.0312452)
Supplement: S1 File — (ZIP) [file pone.0312452.s001.zip › original data for table 1-3 and Fig 1/S1-3_File.pdf]

住院号	高血压病程	DM病程	性别	年龄	血压	
					收缩压	舒张压
	M/Y	M/Y				
ID	高血压病程	DM病程	性别	年龄	收缩压	舒张压
, 00630113	10Y	20Y	女	76	144	74
00994135	N	4Y	男	59	127	82
00967918	2Y	2Y	女	57	129	83
01021603	1M	1M	男	51	134	73
01026748	N	3Y	男	80	153	82
00958325	N	11Y	男	53	137	85
00949182	N	4Y	女	54	134	87
00704680	15Y	11Y	女	60	165	99
,01024971	N	30Y	女	67	148	79
00981103	N	20Y	女	60	130	84
00936555	N	10Y	男	47	120	76
01001432	10Y	10Y	女	69	141	78
00388943	6M	10Y	男	38	163	87
01024980	N	10Y	男	46	104	75
01024949	N	8Y	女	57	107	70
01024601	2Y	20Y	女	66	146	79
01024732	6M	10Y	男	50	164	85
, 00245083	1Y	20Y	女	51	133	74
, 00957835	4Y	10Y	女	56	139	85
, 01018418	2Y	20Y	女	72	128	75
, 00152264	20Y	20Y	男	75	120	73
, 00989214	1Y	26Y	男	66	149	77
, 00717468	N	19Y	女	64	133	77
, 00807530	10Y	2Y	男	48	154	97
, 00523898	N	1Y	男	72	137	72
, 00564022	23Y	8Y	女	72	139	88
, 00234894	10Y	10Y	男	58	129	89
, 01013546	N	1Y	女	44	129	93
, **00723861**	2Y	30Y	男	56	119	69
, 00379955	1Y	13Y	女	46	162	88
, 00981896	2Y	9Y	男	37	137	89
, 01013998	N	20Y	女	71	152	81
, 00962715	N	2Y	男	38	104	73
, 01009150	21Y	1M	女	71	150	85
, 01006092	Y	2Y	男	50	122	76

,	00967041	15Y	15Y	男	51	122	99
,	01006809	Y	16Y	男	47	157	93
,	00984411	N	7Y	男	66	149	93
,	00978270	N	13Y	女	51	137	86
	1009102	Y	Y	女	72	161	87
,	00999092	N	1Y	男	37	102	69
,	00996398	30Y	10Y	女	73	137	82
,	00999940	6M	20Y	女	55	160	86
,	00866757	Y	Y	男	46	137	85
,	00953068	20Y	20Y	女	57	118	79
,	01000409	3Y	3Y	男	41	163	124
,	00308330	N	5Y	男	82	140	80
,	00846626	2Y	8Y	男	56	132	70
,	01002874	N	20Y	女	79	166	91
,	00837909	3Y	5Y	男	42	147	80
,	00960522	2Y	20Y	男	62	138	71
,	00978827	4M	1Y	女	66	157	82
,	00948681	7M	7Y	女	60	158	76
,	00971536	4Y	20Y	男	61	146	79
,	00997786	N	13Y	男	50	133	82
,	00997792	10Y	10Y	女	55	135	96
,	00995718	1Y	10Y	男	38	90	65
,	00724212	N	6Y	女	54	148	97
,	00998057	N	3Y	男	41	134	95
,	00434313	5Y	15Y	女	56	140	86
,	00959754	1Y	10Y	女	64	142	78
,	00993678	N	2Y	男	61	139	59
,	00798086	5Y	20Y	男	62	158	84
,	00276730	6Y	18Y	女	46	109	77
,	00991420	N	15Y	女	54	104	77

G6PD	HbA1c	糖（空腹	血尿酸 U/	血脂四项			
G6PD	HbA1c	糖（空腹	血尿酸 U/	油三酯 T	固醇 CH	度脂蛋白	度脂蛋白
1300- U	4.1-6.5%	-6.1mm	420umo	-2.3umo	-6.5umo	1.91um	-4.14um
G6PD	HbA1c	糖（空腹	血尿酸 U/	油三酯 T	固醇 CH	度脂蛋白	度脂蛋白
2871	6.29	8.74	384	3.65	8.83	1.08	5.55
2331	6.35	4.18	421	1.42	5.18	1.23	3.19
3284	6.8	4.34	230	0.88	4.19	1.26	2.66
2244	11.3	9.71	211	1.69	4.89	0.59	3.34
2893	6.81	10.30	291	3.07	3.18	0.95	1.34
3051	8.99	19.31	246	0.53	4.95	1.92	3.02
2067	6.1	5.03	230	0.51	5.99	2.40	3.57
2371	8.17	8.75	611	3.06	5.04	1.00	2.86
2630	12	17	331	2.38	4.71	0.93	3.03
2942	8.1	6.28	312	1.04	4.96	1.33	3.1
102	5.1	12.54	417	0.82	3.45	1.38	1.82
2206	5.9	5.20	269	2.51	3.95	1.14	2.05
3728	7.24	6.55	385	1.50	5.15	0.74	3.73
3156	5.8	4.4	430	1.94	2.4	0.86	0.66
2329	7.1	6.67	246	2.12	5.15	1.01	3.27
2843	9	2.45	287	0.8	4.22	1.64	2.19
1678	8.98	23.67	591	1.78	6.59	1.45	4.67
1817	6.1	7.12	207	1.59	3.91	1.25	2.09
2565	6.1	5.39	254	1.3	2.69	1.2	0.89
1333	9.41	10.52	332	1.33	6.89	1.45	4.75
3349	6.68	5.80	345	1.45	3.29	0.99	1.74
3026	8.86	7.31	364	1.72	6.27	0.95	4.68
2761	6.32	5.81	341	1.69	4.22	1.15	2.16
2885	5.48	5.06	401	0.81	3.47	1.12	2.09
3395	5.46	5.89	310	1.07	3.46	1.24	1.85
2350	9.28	8.8	401	2.06	3.29	1.12	1.46
2972	8.02	14.58	308	1.21	2.47	0.69	1.34
3525	7	4.57	311	1.52	3.41	1.51	1.47
2282	6.02	3.82	498	2.31	7.18	1.01	4.78
2097	6.6	5.60	285	1.39	5.26	1.58	3.08
1931	7.69	7.35	368	1.24	5.83	0.98	4.19
2866	8.37	8.63	240	1.24	5.29	1.73	2.97
2136	8	6.69	369	0.75	3.42	0.84	2.24
3124	9.5	9.08	254	1.05	3.63	1.44	1.83
3215	10.4	7.09	304	3.16	6.18	1.45	3.58

3130	7.16	5.12	304	1.28	3.95	0.63	2.74
2420	7.21	3.89	540	1.51	4.08	0.92	2.50
4524	6.53	6.00	310	0.89	3.46	1.12	2.06
1908	6.2	4.19	517	0.87	3.15	1.12	1.60
1756	12.5	19.06	292	3.38	6.38	1.32	4.13
2886	5.68	4.89	367	1.73	4.64	0.78	2.85
2658	8.18	5.44	304	2.66	5.87	0.8	3.75
1652	9.5	6.95	480	1.64	4.74	1.07	2.61
2636	10.48	9.18	243	2.5	12.4	1.38	9.07
2454	10.41	6.42	425	0.94	3.36	0.95	1.82
3839	5.95	5.32	483	1.78	5.99	1.26	3.74
2207	8.1	13.44	418	1.6	6.6	1.02	4.28
2623	8	9.51	137	0.56	3.26	1.34	1.47
2539	7.24	6.48	291	1.75	4.60	0.89	2.91
2476	5.29	6.32	310	2.4	5.22	0.81	2.92
2654	8.4	7.24	226	2.01	4.57	0.99	2.79
2130	6.9	14.18	435	1.74	3.24	0.99	1.26
2418	7.07	5.28	452	1.42	5.55	1.61	3.23
2454	5.61	8.15	256	0.53	4.58	1.66	2.31
391	6.73	5.82	227	0.76	2.89	1.17	1.36
2758	6.2	6.66	284	1.97	4.18	1.24	2.09
2836	9.03	7.22	445	1.68	6.87	1.24	4.35
2284	6.2	6.07	345	1.46	8.80	1.64	5.74
3181	6.16	14.86	431	2.89	5.00	0.93	2.93
2216	10.5	3.42	324	1.15	4.93	1.79	2.37
2068	6.8	8.68	286	2.08	4.94	1.02	2.58
2667	5.97	10.23	390	0.70	3.29	1.17	1.72
2681	7.86	6.91	484	2.68	5.85	0.79	3.19
3034	11.94	8.34	319	2.76	11.28	0.99	8.31
2517	10.1	6.85	274	1.26	5.93	1.20	3.96

炎症因子			
IL-6	IL-1β	IL-10	IL-8
< 7	<5 pg/ml	< 9.1	< 62
IL-6	IL-1β	IL-10	IL-8
3.3	< 5.00	< 5.00	5.96
< 1.50	< 5.00	5.0	9.8
2.67	5↑	5.0	17.5
3.73	5↑	5.0	18.4
< 1.50	12.5	5.0	7.39
2.99	< 5.00	< 5.00	7.65
1.62	5↑	5.0	12.7
< 1.50	< 5.00	< 5.00	15.4
1.78	22.3	5.0	15.3
< 1.50	6.44	< 5.00	11
2.93	< 5.00	5.0	11.1
4.22	< 5.00	< 5.00	10.1
10.23	8.22	5.0	14.8
2.59	5↑	5.0	14.9
2.07	5↑	5.0	9.21
2.50	< 5.00	5.0	11.4
1.98	< 5.00	< 5.00	25.9
2.44	5↑	5.0	11.6
7.6	5↑	5.0	9.44
5.55	5↑	5.0	5.93
4.37	< 5.00	< 5.00	27.4
2.94	< 5.00	< 5.00	17.4
2.79	< 5.00	5.0	16.6
2.07	5↑	5.0	9.61
< 1.50	5↑	5.0	5.48
6.79	< 5.00	< 5.00	9.01
1.67	< 5.00	5.11	5.49
< 1.50	< 5.00	5.0	10.3
29.27	5↑	5.0	13.7
2.41	5↑	5.0	24.7
1.86	< 5.00	< 5.00	14.2
< 1.50	5↑	5.0	5.36
2.83	< 5.00	5.0	15.9
< 1.50	5↑	5.0	6.92
< 1.50	5↑	5.0	9.71

2.31	5 ↑	5.0	7.95
4.88	5 ↑	5.0	9.69
4.09	7.16	5.0	12.5
2.96	5 ↑	5.0	11.3
< 1.50	17.7 ↑	5.0	5.55
< 1.50	5 ↑	5.0	14.8
3.24	5 ↑	5.0	8.17
4.67	< 5.00	< 5.00	11.4
10.68	5 ↑	5.0	14.5
7.48	5 ↑	5.0	10.6
< 1.50	< 5.00	< 5.00	15.8
2.8	5 ↑	5.0	6.04
5.89	5 ↑	5.0	12.1
6.37	11.3	5.0	45.2
8.93	5 ↑	7.19	37.2
2.12	5 ↑	5.0	10.5
2.42	< 5.00	< 5.00	12.6
3.32	< 5.00	< 5.00	39.9
4.84	11.4	5.0	12.8
< 1.50	5 ↑	5.0	16.3
2.84	5 ↑	5.0	18.9
< 1.50	5 ↑	5.0	5.36
< 1.50	5 ↑	5.0	5.0
2.18	5 ↑	5.0	22.5
< 1.50	5 ↑	5.0	7.75
1.5	5.0	5.0	48.3
< 1.50	5 ↑	5.0	5.58
4.03	11.1	5.0	17
2.69	5 ↑	5.0	19.9
< 1.50	< 5.00	< 5.00	15.2

	肝功能			肾功能			
TNF-α	丙转氨	草转氨	胆红素	尿素氮	肌酐	小球滤过	钾
＜8.1	0-40U/L	0-45U/L	-20umo	-7.5mm	133mm	min·1.73	-5.5mm
TNF-α	丙转氨	草转氨	胆红素	尿素氮	肌酐	小球滤过	钾
10.4	10.5	15.9	5.3	6.4	91	52.56	4.42
6.3	31	26	3.8	5.29	58	105.82	4.71
6.88	12	12	9.8	3.92	46	106.4	4.31
9.05	7	11	5.2	4.68	88	86.76	3.96
9.96	31.1	25.2	16.6	5.4	62	88.5	3.97
7.28	10.5	23.7	10.5	4.1	64	106.5	4.13
5.74	14.9	18.1	10.3	5.3	52	104.24	3.88
8.90	11.1	20.4	5.3	11.5	97	54.7	3.55
8.47	17	20	7.5	5.4	45	99.9	3.91
7.29	14.7	13.5	5.6	6.1	53	100.09	4.26
9.26	52	30.2	7.9	6.1	86	92.58	3.98
6.85	14	14	11.7	4.3	54.1	93.45	3.45
21.8	12	8	4.8	17.14	771	6.93	4.30
14.6	19.4	24	3.6	9.4	144	49.78	4.87
5.57	14.2	11.5	8.4	6.6	34	117.97	4.31
7.22	9	9	6.4	3.11	42	102.55	4.26
16.5	18.9	17.7	3.4	27.7	565	9.28	4.15
6.34	15	21	6.5	5.15	22.2	141.22	4.15
9.86	15	17	10.7	4.75	63	95.52	4.54
5.68	15.8	19.2	8.9	5.6	62	86.23	5.01
10.3	47	38	16.8	5.1	77	88.77	4.12
11.4	11	12	19.9	8.86	84	83.08	3.92
7.75	96.6	71.1	15.2	5.5	54	96.11	4.43
5.79	13	15	10.9	6.35	79	101.11	4.01
7.26	20	19	12.2	6.84	64	92.54	3.83
9.09	46	43	5.0	7.27	85	58.86	3.37
9.84	18.6	16.2	19.5	9	157	41.13	3.14
5.30	18.1	15.4	9.0	4.3	54	110.61	3.97
14.8	16	17	6.1	26.55	1559	2.61	3.98
18.1	55	48	6.3	8.58	141.6	38.37	5.22
11.9	25	15	15.3	5.86	55	126.72	3.84
7.96	11	12	6	3.85	45	97.05	4.10
7.38	26.4	15.8	13.1	4.3	76	110	3.74
6.86	29	27	7.4	6.1	58	89.16	4.30
7.63	13	13	5.9	8.76	93	82.37	4.50

7.43	25.8	18.7	14.0	3.9	59	112.04	3.84
12.2	11	15	6.1	12.4	222.4	29.19	5.59
8.91	18	20	52.0	7.42	52	105.50	3.57
7.97	65	54	3.1	5.32	77	77.38	4.71
7.17	14	15	14.2	12.2	51	91.73	4.57
6.08	11	13	12.9	5.23	74	111.86	4.08
11.4	23.5	23.5	7.6	5.83	75	67.71	4.07
7.98	10.3	15.4	3	11.9	127	40.91	3.96
7.18	28	21	3.3	4.58	97	80.08	3.98
8.19	13	38	11.4	5.36	53.8	100.96	4.00
9.52	29	19	19.6	4.86	75	108.23	3.16
9.70	32.2	26.6	6.2	6.2	80	78.71	4.24
16.3	10	5	7.3	27.6	895	5.10	5.21
16.1	15	18	8.6	7.75	59	83.28	4.54
16.1	25.4	24.3	3.1	20.6	421	14.01	3.85
7.82	13.7	10.8	6.0	6.2	73	94.30	4.18
8.75	14.8	212	3.1	9.5	92	56.12	4.53
10.6	7	14	3.1	16.63	180	25.92	3.51
11.8	48.6	27.5	9.8	14.7	666	7.04	4.88
7.75	19	21	20.9	4.06	67.4	106.38	3.84
6.56	10	14	6.9	8.45	74.3	78.49	4.23
10.5	20	13.3	4.5	20.4	124	63.29	4.76
7.59	12.2	16.6	5.4	7.8	123	43.00	4.28
4	16	12	10.1	5.42	89	91.96	3.79
7.9	14	17	6.2	6.85	108	49.64	3.67
10.3	15	64	2.3	34.2	317	12.73	4.5
6.88	16	20.9	5.7	5.4	86	83.91	4.9
10.2	19	12	1.3	17.97	147	43.52	4.49
6.79	8.7	8.1	4.4	6.2	77	80.14	3.59
7.08	8	11	3.3	4.30	45	109.36	3.71

电解质5项								
钠	氯	钙	O2结合	磷	总蛋白	1清白蛋	球蛋白	接胆红
-145mm	110mm	-2.55mn	-34mmo	3-1.5mmol				1.1
钠	氯	钙	O2结合	磷	总蛋白	1清白蛋	球蛋白	接胆红
139	102	2.52	24.5					2.3
145.2	105.2	2.29	26.8	1.09	71.8	44.2	28	
143.5	106.7	2.28	25.4	1.38	64.6	43.9	21	
141	103.8	2.20	26.4	1.05	70.6	36.3	34	2.61
137	101.2	2.32	23.4					5.8
136	97.5	2.36	25.9					3.7
141	105.0	2.44	26.7					3.6
139	101.4	2.42	24.5					2
143	104.7	2.45	24.1					2.9
136	99.4	2.37	28.4					2.4
138	105.6	2.25	20.8					3.1
143	107.8	2.19	25.7	1.15	62.3	39.3	23	
139.8	100.8	2.02	24.4	2.17	60.9	36.6	24	
142	109.8	2.43	20.5					1.7
140	103.9	2.40	26.7					2.9
143	105.7	2.29	27.1		65.2	42.2	23	
136.9	100.7	2.01	22.2					1
144	103.8	2.26	28.7					
142.3	105.3	2.29	24.9					
137	100.5	2.47	26.1					
138	100.9	2.52	23.2					
143.2	104.8	2.25	23.9	1.12				
139	99.7	2.52	25.2					5.5
143	104	2.3	25.1	1.24				
142.3	105.5	2.23	24.1	1.05				
142	102.7	2.48	26.1					
142	106.6	2.37	23.4					
139	104.4	2.45	25.8					3.4
125.9	84.3	1.92	22.9	2				
140.3	107.3	2.34	26.9	1.54				
144	105.4	2.35	23.2	1.36	70.8	47.5	23	
141.3	104.9	2.31	25.0	1.43	72.4	44.5	28	
140	103.9	2.41	24.7					4.5
140	104.0	2.42	23.4					
142.5	106.7	2.11	23.9	1.39				

139	104.6	2.24	24.8					
141.8	111.9	2.22	21.1					
141.6	105.9	2.11	19.7	0.94				
143.4	111.0	2.26	23.5	1.52				
134	99.1	2.63	26					
142.1	100.0	2.34	26.9	1.26				
141.3	106.4	2.26	24.9					
139	105.9	2.13	23.8					
139.8	105.4	1.99	23.7	1.05				
139.1	104.7	2.26	27.3	1.26				
142.5	102.1	2.35	30.9	0.93	73.3	47	26	
136	103.2	2.15	22.7					1.8
138	101.3	2.15	20.5	1.78	63.6	40.1	24	
141	103.0	2.35	25.6	1.26	67.6	42.7	25	
139	106.5	2.00	22.4					1.4
143.1	105.4	2.27	26.4					2.4
136	103.2	2.26	23.9					1.7
142.7	103.4	2.05	28.1	1.55	53.7	29.1	25	0.99
138	101.3	2.17	22.8					4.3
142	104.3	2.31	29.6	1.44				
140	99.8	2.63	30.0	1.2				
132	98.1	2.39	27.4					
141	106.7	2.35	25.2					
141.8	103.9	2.20	27.0	1.1	66.1	44.5	22	
142.1	103.9	2.38	25.4	1.41	74.8	48	27	2.42
140.4	106.5	2.1	21.4	1.53	61	36.9	24	1.07
138.6	103.8	2.28	26.7					3
142.3	105.4	2.22	22.6	1.67	56.9	30.6	26	
141.9	102.7	2.24	29.1					
140	102.8	2.18	26.8	1.52				

C肽

.-4.4 ng/ml

C肽

0.55
1.95
1.74

1.52

1.31

0.31
