# Supplementary figures and images for: Activity of glucose-6-phosphate dehydrogenease and its correlation with inflammatory factors in diabetic retinopathy
Source: PLoS One. 2024 Dec 2;19(12):e0312452. doi: 10.1371/journal.pone.0312452 (PMC11611225; doi:10.1371/journal.pone.0312452)

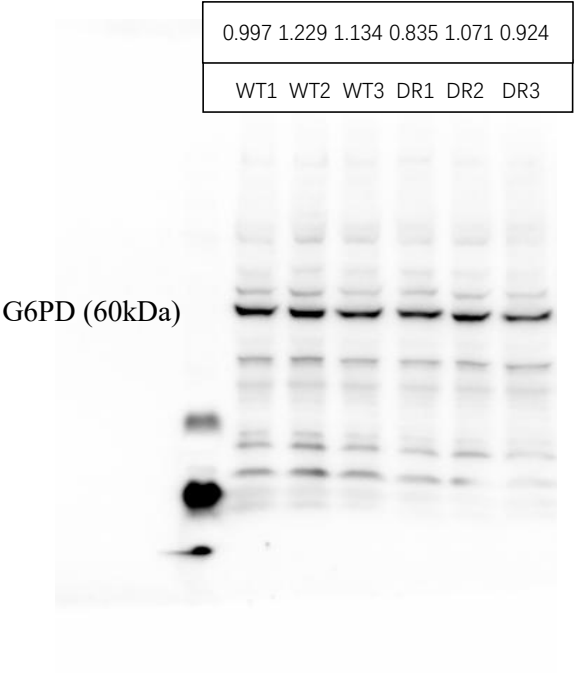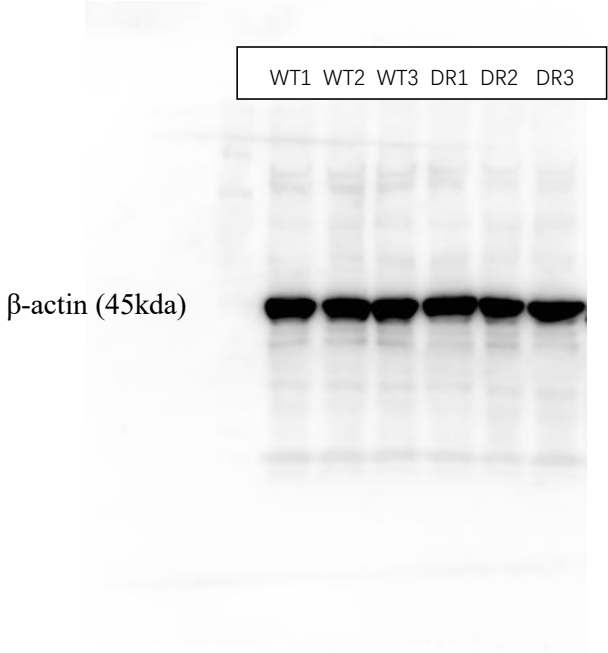

Supplement: S1 File — (ZIP) [file pone.0312452.s001.zip › S1_raw_images.pdf]
