# Supplementary material for: Activity of glucose-6-phosphate dehydrogenease and its correlation with inflammatory factors in diabetic retinopathy
Source: PLoS One. 2024 Dec 2;19(12):e0312452. doi: 10.1371/journal.pone.0312452 (PMC11611225; doi:10.1371/journal.pone.0312452)
Supplement: Attachment — Submitted filename: PONE-D-23-43875.docx [file pone.0312452.s002.docx]

**Understanding obesity-associated insulin resistance and immune response through the lens of evolutionary medicine**

This paper explores single-cell assay for transposase-accessible chromatin sequencing and single-cell ribonucleic acid sequencing (scRNA-seq) to evaluate lambdoidal junction tissue (formed by the maxillary, and lateral and medial nasal processes) from C57BL/6J mouse embryos at 10.5, 11.5, and 12.5 embryonic days of development. Authors identified relevant subgroups of mesenchymal and ectodermal cells involved in the fusion process and characterized cell-type specific transcriptional factors and performed cell differentiation trajectory analysis, and found functional genes such as Nfix, Sox1, Sox3, and Hes5 to be highly activated, this potentially facilitated the transition of relevant cell types. Additionally used scATAC data and found key genes for the fusion process to demonstrate differential chromatin accessibility among major cell types. The authors established map-based cloning and molecular design breeding in rice.

However, I have few concerns on current manuscript:

1. The authors integration of scATAC-seq and scRNA-seq done by Seurat package. However, the authors missed the version of Seurat package. Update the version of Seurat.

2. Authors identified relevant subgroups of mesenchymal and ectodermal cells involved in the fusion process, and characterized cell-type specific transcriptional factors involved in failure of fusion at this seam causes cleft lip and/or primary palate (CL/P). What are the other cell types and their genes regulator’s role in cleft lip and/or primary palate (CL/P)?

3. In this study authors identified Brax1, Sox2, and Ahr genes that may be involved in maintaining their functions. What is their gene expression profiles in three conditions of samples. Can authors make Vlnplot for three conditions of genes expression profile (day 10.5, 11.5, and 12.5 embryos)?

4. Did authors identify any novel genes and transcription factors of cleft lip and/or primary palate (CL/P)?

This manuscript is good in shape and well-established objectives of this study. I’m happy to accept this manuscript after minor corrections.
