# Supplementary material for: Activity of glucose-6-phosphate dehydrogenease and its correlation with inflammatory factors in diabetic retinopathy
Source: PLoS One. 2024 Dec 2;19(12):e0312452. doi: 10.1371/journal.pone.0312452 (PMC11611225; doi:10.1371/journal.pone.0312452)
Supplement: Attachment — Submitted filename: Response to Reviewers.docx [file pone.0312452.s003.docx]

**Response Letter**

Dear editor and reviewers,

We would like to express our sincere appreciation for your letter and the reviewers’ constructive comments concerning our article entitled “Activity of glucose-6-phosphate dehydrogenease and its correlation with inflammatory factors in diabetic retinopathy” (Manuscript No: PONE-D-24-26543). We have revised our manuscript accordingly. In this revised version, changes to our manuscript are highlighted within the document using red-colored text. Point-by-point responses to the reviewers’ comments are listed below. Once again, we appreciate your kind consideration of our submission.

With lots of thanks,

Dan Liu

**Response to reviewer #1**

**Comment 1:** Can you explain why you did a clinical assessment of glucose-6-phosphate dehydrogenase (G6PD) activity in DR and its correlation with inflammatory factors and again an experiment in rats?

**Response:** *We welcome the reviewer's careful reading and questions. The pathogenesis of diabetic retinopathy (DR) is diverse, and G6PD is a key enzyme in sugar metabolism and oxidative stress mechanisms, playing an important role in DR development. The oxidative stress pathway cannot function without inflammatory factors, participants in the same pathway. Therefore, we aimed to study their relationship to investigate DR pathogenesis. Through clinical trials, we found a certain correlation between the two. Moreover, from the glucose metabolism perspective, the occurrence and development of DR study is insufficient, with most research limited to clinical epidemiological surveys. To explore if the pentose phosphate pathway, a key branch of glucose metabolism, may causally regulate DR occurrence and development, we constructed a STZ-induced diabetic rat model and analyzed the correlation between G6PD and related inflammatory factors throughout the process.*

**Comment 2:** Kindly add the clinical significance of your work.

**Response:** *Thank you for your suggestions. Exploring the causal relationship between G6PD and DR (diabetic retinopathy) could provide new insights and treatment directions. We have added a discussion of the clinical significance of this study in the article's discussion section, highlighted in red font. Please refer to lines 244-250 of the article.*

**Response to reviewer #2**

**Comment 1:** The study provides cross-sectional data which is valuable, but the lack of longitudinal follow-up limits the ability to observe the progression of DR and the long-term impact of G6PD activity.

**Response:** *We would like to express our deepest gratitude for your insightful feedback, which has played a pivotal role in enhancing the quality of our research. In the discussion section of the article, we have meticulously acknowledged the inherent limitations of cross-sectional studies and have provided a comprehensive set of relevant optimization suggestions to mitigate these drawbacks. You can find these detailed insights in the lines 291-298 of the article.*

**Comment 2:** While the sample size is adequate for the current study, a larger sample could enhance the generalizability of the findings.

**Response:** *We greatly appreciate your comments and suggestions. We have acknowledged the limitation regarding sample size in the article and provided relevant solutions. Please refer to the discussion section of the article for details.*

**Comment 3:** The study does not extensively address potential confounding factors, such as diet, medication, or lifestyle, which could influence both G6PD activity and DR progression.

**Response:** *Thank you for your comments. In experimental research, confounding factors can indeed affect the results, so controlling these factors is crucial. Therefore, we aim to eliminate or control confounding variables during the experimental design phase and use appropriate statistical methods during the data analysis phase to adjust for the impact of these factors. This approach helps improve the reliability and validity of the research findings. We are aware of this limitation and have added a brief discussion of these potential confounding factors and their possible impact on the results in the article, as detailed in lines 256-260.*

**Comment 4:** The study establishes correlations but does not delve deeply into the mechanistic pathways linking G6PD activity to DR.

**Response:** *Thank you for your valuable suggestion. Hypoactive or hyperactive G6PD has been linked to the pathology of type II diabetes, but there are no clear reports on the mechanisms by which G6PD influences the occurrence and progression of DR (diabetic retinopathy) currently. In endothelial cells, it is now clear that G6PD would modulate vascular endothelial growth factor and represent a novel angiogenic regulator. As a chronic inflammatory disease, inflammatory factor generation might also play a pivotal role in the pathogenesis of DR. The interaction between G6PD and inflammation factors remains an area for further investigation. Therefore, we hypothesize that G6PD may affect the progression of DR through modulating the production of inflammation factors such as IL-6, IL-8, TNF-α. As the first enzyme of the pentose phosphate pathway, which is the principal intracellular source of NADPH, G6PD may affect these inflammation factors through a series of complex oxidative stress signal pathway, which will be a huge challenge to elucidate clearly. Therefore, we have added possible mechanisms and future research plans in the discussion section, as detailed in lines 263-286 of the article.*

**Comment 5:** The statistical models used are comprehensive, but their complexity might make the analysis less accessible to a broader audience.

**Response:** *We greatly appreciate your comments. We have added explanations related to statistical methods in the methodology section to help readers better understand the article.*

**Comment 6:** The manuscript hints at the therapeutic potential of modulating G6PD activity but does not explore this in depth.

**Response:** *Thank you for your careful reading and suggestions. Following your suggestion we have included the potential therapeutic significance of G6PD and specific future research directions in the article. Please refer to lines 234-250 for the details.*

**Comment 7:** There are a few minor typographical errors and inconsistencies in formatting throughout the manuscript.

**Response:** *Thank you for your careful review. We apologize for our oversight. Based on your feedback, we have thoroughly proofread and formatted the article.*

**Comment 8:** The figures and tables are generally well-prepared, but some could benefit from additional labeling or explanations to enhance clarity.

**Response:** *Thank you for your comments. We have rechecked all the figures in the article to ensure that each one has proper labels and necessary annotations.*

**Comment 9:** The ethical approvals for both human and animal studies are well-documented, but a more detailed discussion of the ethical implications of your findings would strengthen the manuscript.

**Response:** *Thank you for your feedback. We have incorporated ethical considerations regarding the potential translation of this research into clinical practice, as suggested. Please refer to lines 244-250 of the article for details.*

**Comment 10:** The manuscript references relevant literature, but the discussion could benefit from a more critical comparison with previous studies.

**Response:** *Thank you for your suggestion. We have added a discussion of the current controversies and consensus in related research to the discussion section of the article, and we have also detailed how our study contributes to and impacts the current research field.*
